# Research priorities in childhood-onset lupus: results of a multidisciplinary prioritization exercise

**DOI:** 10.1186/s12969-019-0327-4

**Published:** 2019-07-01

**Authors:** Stacy P. Ardoin, R Paola Daly, Lyna Merzoug, Karin Tse, Kaveh Ardalan, Lisa Arkin, Andrea Knight, Tamar Rubinstein, Natasha Ruth, Scott E. Wenderfer, Aimee O. Hersh, on behalf of the Childhood Arthritis and Rheumatology Research Alliance and Lupus Foundation of America

**Affiliations:** 1 Division of Pediatric Rheumatology, Nationwide Children’s Hospital, 700 Children’s Drive, Columbus, OH 43205 USA; 2 Lupus Foundation of America, Washington, DC USA; 3 Ann & Robert H. Lurie Children’s Hospital, Chicago, IL USA; 4 University of Wisconsin, Madison, WI USA; 5 Hospital for Sick Kids, Toronto, Ontario Canada; 6 Children’s Hospital at Montefiore, Bronx, NY USA; 7 Medical University of South Carolina, Charleston, SC USA; 8 Texas Children’s Hospital, Houston, TX USA; 9 University of Utah, Salt Lake City, UT USA

**Keywords:** Lupus, Pediatric, Research, Nephritis, Neuropsychiatric, Cutaneous

## Abstract

**Background:**

Childhood-onset systemic erythematosus lupus (cSLE) is characterized by more severe disease, widespread organ involvement and higher mortality compared to adult-onset SLE. However, cSLE is largely underfunded to carry out necessary research to advance the field. Few commonly used SLE medications have been studied in children, and important knowledge gaps exist concerning epidemiology, genetics, pathophysiology and optimal treatments for cSLE.

**Methods:**

In order to assess highest cSLE research priority areas, the Lupus Foundation of America (LFA) and Childhood Arthritis and Rheumatology Research Alliance (CARRA) administered a cSLE research prioritization survey to pediatric rheumatologists, dermatologists and nephrologists with expertise in lupus. Members of LFA and CARRA’s SLE Committee identified a list of cSLE research domains and developed a 17-item tiered, web-based survey asking respondents to categorize the research domains into high, medium, or low priority areas. For domains identified as high priority, respondents ranked research topics within that category. For example, for the domain of nephritis, respondents ranked importance of: epidemiology, biomarkers, long-term outcomes, quality improvement, etc. The survey was distributed to members of CARRA, Midwestern Pediatric Nephrology Consortium (MWPNC) and Pediatric Dermatology Research Alliance (PeDRA) Connective Tissue Disease group.

**Results:**

The overall response rate was 256/752 (34%). The highest prioritized research domains were: nephritis, clinical trials, biomarkers, neuropsychiatric disease and refractory skin disease. Notably, nephritis, clinical trials and biomarkers were ranked in the top five by all groups. Within each research domain, all groups showed agreement in identifying the following as important focus areas: determining best treatments, biomarkers/pathophysiology, drug discovery/novel treatments, understanding long term outcomes, and refining provider reported quality measures.

**Conclusion:**

This survey identified the highest cSLE research priorities among leading rheumatology, dermatology and nephrology clinicians and investigators engaged in care of children with lupus. There is a strong need for multidisciplinary collaboration moving forward, which was indicated as highly important among stakeholders involved in the survey. These survey results should be used as a roadmap to guide funding and specific research programs in cSLE to address urgent, unmet needs among this population.

## Background

Childhood-onset systemic erythematosus lupus (cSLE), defined as onset of SLE at age < 18 years, affects an estimated 6000 US children and adolescents [1, 2]. Approximately 20% of individuals with SLE develop the disease in childhood. International studies indicate that children with cSLE have more pervasive and life-threatening organ involvement than adults. Up to 80% of children with cSLE develop lupus nephritis (LN), resulting in higher mortality rates in childhood versus adult-onset SLE [3]. Healthcare costs for children and adolescents are high, estimated to be on average $1500 per patient per year [4]. No medication has been approved by the Food and Drug Administration (FDA) specifically for children and adolescents with cSLE. Thus, treatment of cSLE is generally extrapolated from clinical trials performed in adults with SLE. While many aspects of adult and childhood SLE pathophysiology are likely similar, important differences exist concerning genetic predisposition, environmental triggers, pharmacokinetics and management of concerns specific to the pediatric population such as neurodevelopment, growth, puberty, mental health and educational development [5]. Our understanding of cSLE is limited by key gaps in knowledge surrounding these factors, which influence natural history as well as the identification of phenotypic and molecular heterogeneity impacting treatment choices, clinical outcomes and medication toxicity.

To address these knowledge gaps in cSLE, the Lupus Foundation of America (LFA) and Childhood Arthritis and Rheumatology Research Alliance (CARRA) partnered to explore and better understand clinicians’ and investigators’ main research priorities for children with lupus which should guide future research decisions and funding mechanisms. Lupus as a systemic disease requires coordination across specialties, and a research prioritization exercise – never conducted in this audience of cSLE experts – reflects the actual complex nature of the disease and will provide an evidence base to guide future research decisions. CARRA is well-suited for this exercise as a research network representing approximately 90% of North American pediatric rheumatologists. CARRA’s work products have included the development of consensus treatment plans for proliferative lupus nephritis in children [6] and other publications related to gaps in care and research in cSLE and other rheumatic diseases [7–13]. CARRA’s SLE Committee also has existing research partnerships with nephrologists through the Midwestern Pediatric Nephrology Consortium (MWPNC) and dermatologists through the Pediatric Dermatology Research Association (PeDRA). In order to capture a multidisciplinary perspective on cSLE research priorities, a survey was developed and administered to pediatric clinicians and investigators in rheumatology, nephrology and dermatology.

## Methods

### Ethics approval

This study was determined not to be human subjects research by the Institutional Review Board at Nationwide Children’s Hospital.

### Survey instrument

The survey instrument was developed by a working group that included members of CARRA, MWPNC, PeDRA and the LFA. The principal items of the survey were identified via literature review, clinical expertise and review of a previous unpublished consensus exercise in this area and served as the framework for the first iteration. The research domains represented the following broad categories: organ specific lupus manifestations (for example, nephritis, neuropsychiatric disease, and refractory skin disease), clinical management (for example, mental health, transition to adult care, reproductive health) and research approaches (for example, clinical trials, biomarkers/pathophysiology, and quality of life).

**Fig. 1:**
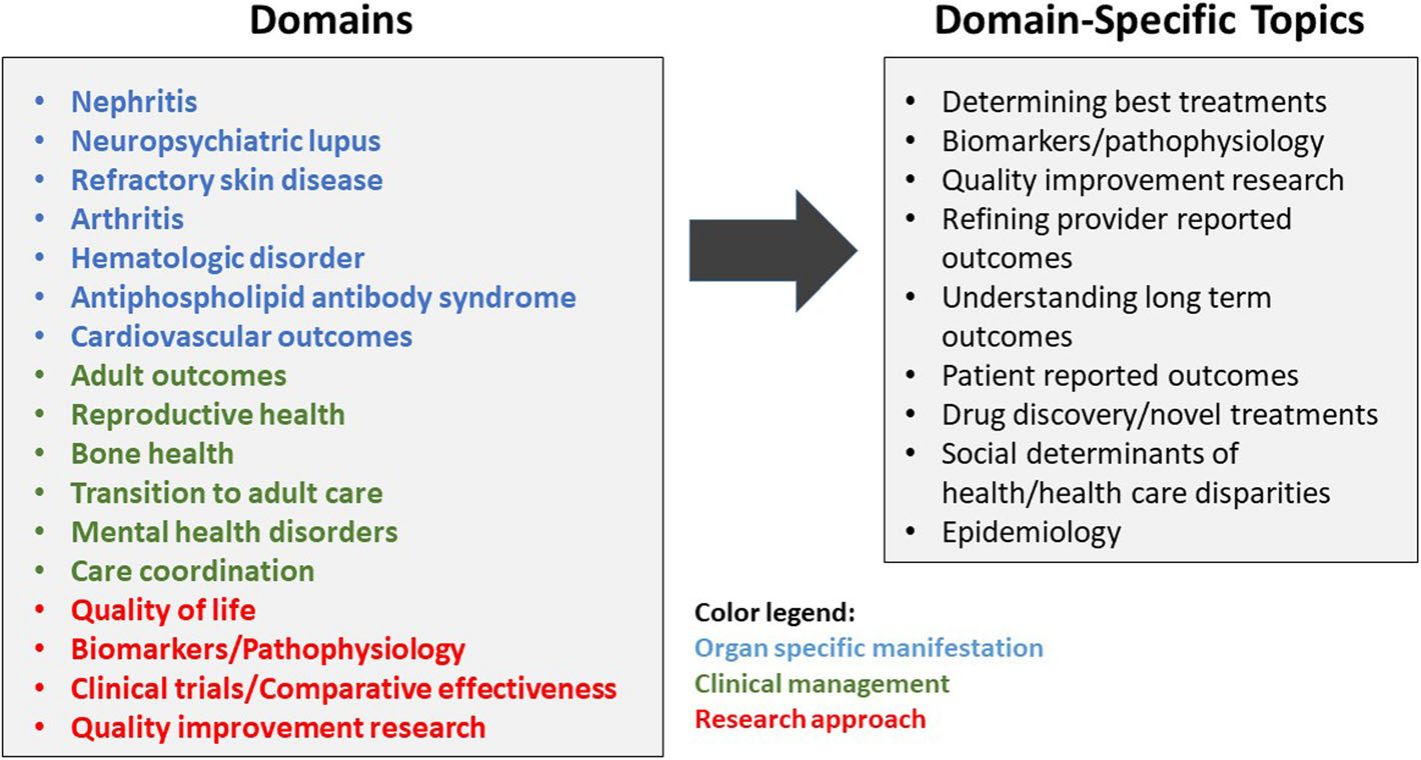
Respondents were first asked to rank the 17 research domains as high, medium and low priority. Research domains were categorized according to organ specific manifestations (blue), clinical management (green) and research approaches (red). Next, for those domains ranked as high priority, respondents were asked additional questions about research topic priorities within that domain

Working group members proposed a two-level design for the survey. In the first level, respondents ranked 17 research domains as high, medium or low priority for cSLE research. Respondents were then asked to rank research topics for each domain marked as high priority (see Fig. 1). Of the 17 research domains, second level prioritization was available only for organ specific and clinical management research domains. Subsequent iterations of the instrument involved the standardization of response choices for the second level of prioritization to allow comparison across domains and respondent groups. Following development of the survey, the instrument was tested for comprehension and general appeal by 25 individuals of the intended audience. The survey was finalized with received feedback and tested for quality control of technical aspects.

### Survey administration

The survey was distributed by email through a©2018 Qualtrics survey link between May and August 2018 to 752 members of CARRA, MWPNC and PeDRA. Each group distributed electronic survey links to its membership and electronically sent reminders to non-responders at varying intervals, dependent on the group’s usual communication practices. CARRA and MWPNC shared the survey link with all of their members while PeDRA shared the survey with members of its Connective Tissue Disease Group. Two mid-course modifications were made to the distribution communications in response to feedback received. First, the invitation language was edited to note that individuals who felt they did not have sufficient lupus expertise could opt out of the survey. Second, additional reminders were sent to CARRA members belonging specifically to the CARRA SLE Committee to increase response rates of clinicians and investigators who primarily treat cSLE.

### Survey data analysis

Descriptive data was generated through Qualtrics, and additional sub-analysis was completed in IBM SPSS Statistics Version 25. Kruskal-Wallis and post-hoc pairwise comparison tests were used to compare first-level and second-level rankings between CARRA, MWPNC and PeDRA.

## Results

The overall response rate for the survey was 256/752 (34%). The CARRA membership response rate was highest with 174/403 (43%) of respondents completing the survey, and 150/174 (86%) of those respondents identified as members of the CARRA SLE Committee. The MWPNC membership response rate was 66/297 (22%) and for the PeDRA Connective Tissue Disease Group, 16/52 (31%). By specialty, 55% identified as pediatric rheumatologists, 24% as nephrologists, 7% as dermatologists, 2% as adult and pediatric rheumatologists, 9% as fellows, and 2% as “other.”

**Fig. 2:**
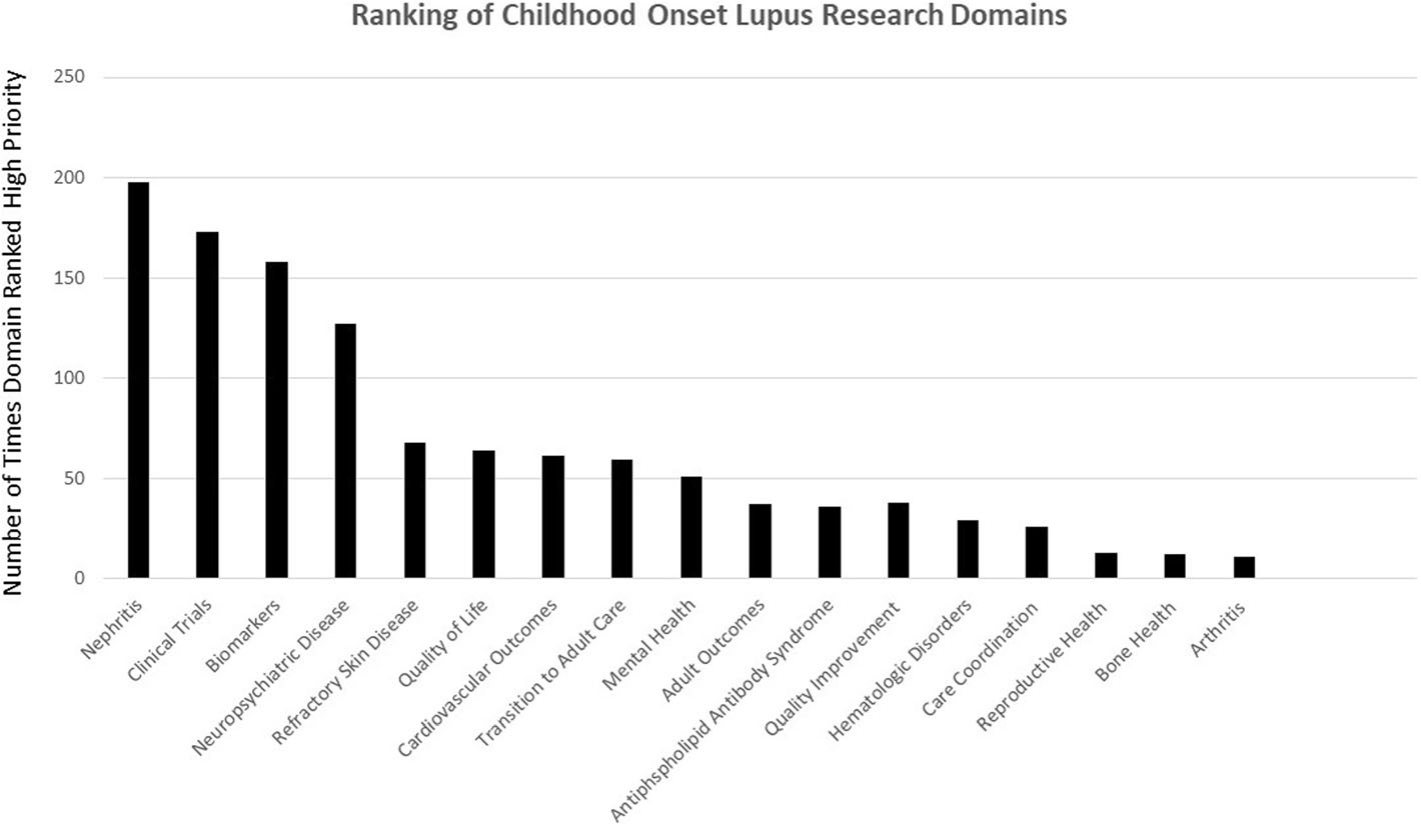
Shown are the number of survey respondents who ranked each domain as a high research priority

**Fig. 3:**
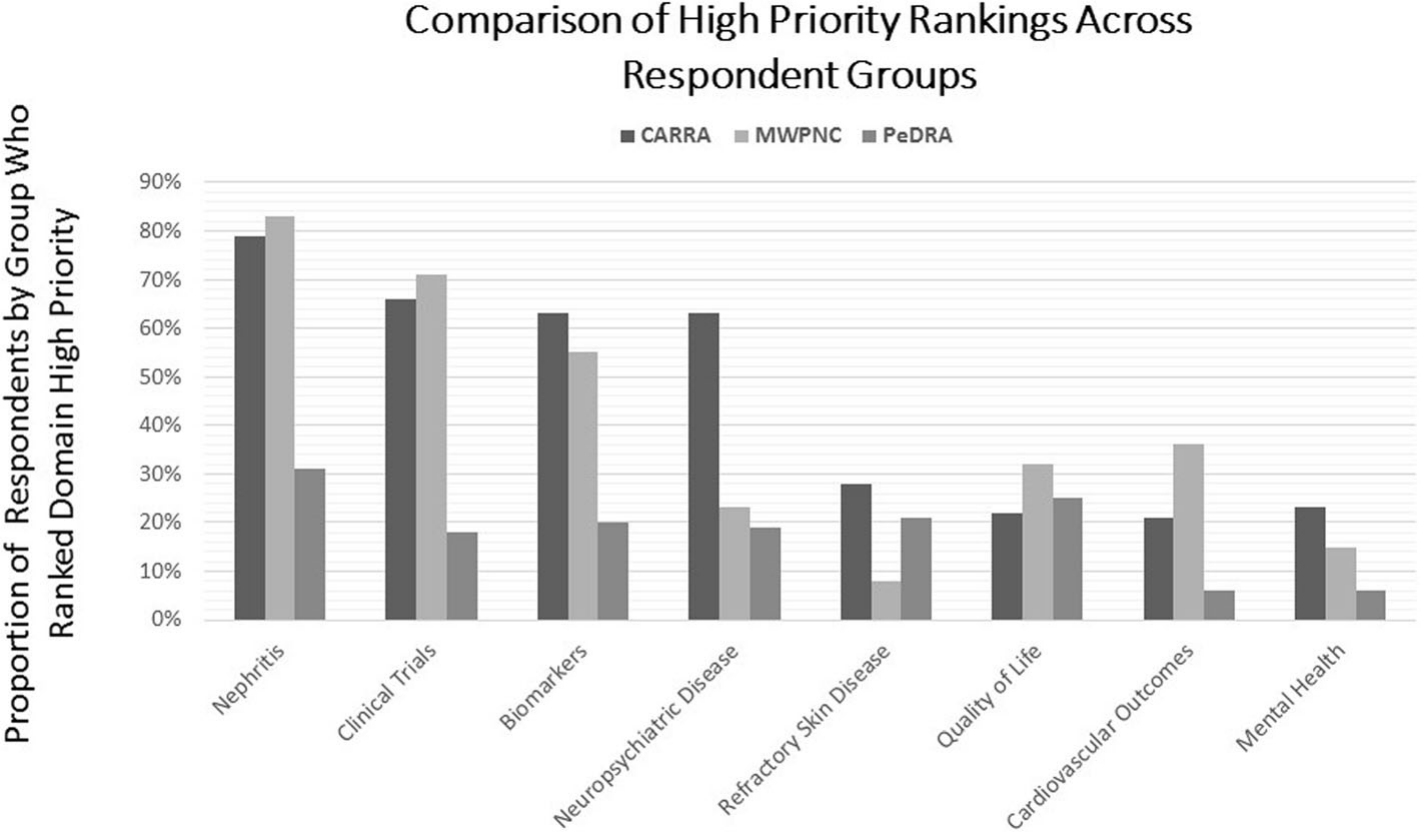
Depicts top ranking research domains by organization, including each organization’s top five rankings. For CARRA, number of respondents = 174; MWPNC, 66; PeDRA, 16

Research domain rankings are summarized in Fig. 2. Across all specialties, the top five ranked research domains included nephritis, clinical trials, biomarkers, neuropsychiatric disease and refractory skin disease. Figure 3 depicts the five highest ranking research domains for each organization (CARRA, MWPNC, and PeDRA). CARRA responses mirror the results of the overall ranking of top five research domains, with slight differences among PeDRA and MWPNC respondents. PeDRA respondents agreed with the four priority research domains of nephritis, clinical trials, biomarkers and refractory skin disease but also included quality of life instead of neuropsychiatric disease. MWPNC also included quality of life and cardiovascular outcomes, rather than neuropsychiatric disease and refractory skin disease. CARRA members were significantly more likely to rank neuropsychiatric disease as a top research domain compared to MWPNC (*p* = .003). Not surprisingly, nephrologists were significantly more likely to rank nephritis as the highest research priority compared to CARRA (*p* = .045), and dermatologists were more likely to rank refractory skin disease as highest priority compared to CARRA (*p* = .001).

Looking at the second level of prioritization for the top two research domains, respondents indicated the five most important research topics for both nephritis and neuropsychiatric disease were: determining best treatments, biomarkers/pathophysiology, drug discovery/novel treatments, understanding long term outcomes, and refining provider-reported clinical measures. Sub-analysis for nephritis showed nephrologists were more likely to rank understanding long-term outcomes higher than CARRA members (*p* = .016), however all groups concurred that long-term outcomes are among top research needs under nephritis. For neuropsychiatric disease, there were no statistically significant differences between specialties in how the top five research topics were ranked. Overall, the main research topics identified were consistent across CARRA, MWPNC and PeDRA respondents for these two domains. Of note, research on understanding long-term outcomes was identified in the top five priorities across all research domains. Further research on biomarkers/pathophysiology and determining best treatments were also indicated as high priority in most domains.

Respondents were asked which sub-specialties in addition to rheumatology should collaborate in cSLE research. Overwhelmingly, 215/234 (92%) of respondents suggested collaborating with nephrologists and 171/234 (73%) suggested dermatologists. Although mental health was not reported among the highest ranking research domains, 158/234 (68%) still suggested collaborating with mental health specialists. Neurologists and immunologists were also among the top five specialists respondents believe should be involved in cSLE research (135/234, 58%, 130/234, 56%, respectively).

## Discussion

In an effort to determine multidisciplinary research priorities in cSLE, CARRA and LFA partnered to survey leading clinician and research stakeholders involved in the care of children and adolescents with lupus: rheumatologists (CARRA members), nephrologists (MWPNC members) and dermatologists (PeDRA members). The 34% response rate is robust for this type of survey with representation from all groups and is comparable to similar CARRA surveys related to lupus and other rheumatic diseases [14–18]. Nephritis, clinical trials, biomarkers, neuropsychiatric disease and refractory skin disease emerged as the highest priority cSLE research domains. Looking at the two most highly ranked research domains of nephritis and neuropsychiatric disease, responses showed a clear prioritization of the following topics: determining best treatments, biomarkers/pathophysiology, drug discovery/novel treatments, refining provider reported clinical outcomes and understanding long term outcomes.

These results confirm critical, unmet needs in cSLE. It has been well established that lupus nephritis is highly prevalent in cSLE, and despite clear advances in the improvement of patient and renal survival over the last several decades, clinicians and investigators across specialties agree further research needs to be carried out, particularly around treatments and outcomes. Treatment for cSLE, and particularly LN, is extrapolated from adult clinical trials data, and optimal drug dosing, duration of therapy and outcomes measurement may be distinct for pediatric patients. Of the 29 studies actively recruiting participants for LN studies cited at clinicaltrials.gov, only 10 include children [19]. It is important to note that while these trials include children, drawing statistically significant results is difficult if there are not enough enrolled children compared to adults. Most of these active trials also exclude younger children, as age eligibility criteria begins at 14 or 16 years of age and extends into adult populations. Additionally, studies have identified candidate serum and urinary biomarkers in childhood LN, yet none have achieved widespread clinical use [20, 21]. Determining and discovering best treatments, along with biomarker research, are among top priorities outlined in this survey and are clearly lacking in current lupus research.

Neuropsychiatric lupus remains a very challenging topic for clinicians and researchers, particularly given the heterogeneity of manifestations, challenges in diagnosis and very limited ability to achieve histologic confirmation of tissue involvement. Only one actively recruiting clinical trial for neuropsychiatric lupus is currently listed at clinicaltrials.gov, and it does not include children. Most published research focuses on identification of biomarkers and central nervous system imaging, which is in line with the findings of the significance of biomarkers for neuropsychiatric lupus. Injury to the central nervous system can have substantial negative impact on the neurodevelopment, educational and vocational outcomes in children and adolescents, and further research in this area is desperately needed to characterize long-term outcomes and treatment [22, 23].

Limitations of this study include potential self-selection bias from using a convenience sample as only members of CARRA, MWPNC and PeDRA were included in the sample. These groups are most prominent in clinical care and research of cSLE, and agreement among this group was the desired outcome. These organizations include individuals from various specialties with broad research experience including psychologists, neurologists, immunologists, basic scientists, geneticists and bioinformaticians. Some important collaborators are not represented in this survey (for example, pathologists, radiologists) and may be included in future work. Additionally, there were a small number of PeDRA respondents as PeDRA leadership determined it would be most appropriate to survey only members of the Connective Tissue Disease study group rather than the entire membership; this workgroup focuses on treating and studying connective disease diseases and other autoimmune disorders such as cutaneous lupus. The patient and family voice was not directly captured with this survey, but including these stakeholders is planned in future work using focus groups and different survey methodology. Some of the research domains were broad andhad content overlap (for example, adult outcomes and cardiovascular outcomes), but the intention was to be inclusive of potential research domains. A particular strength of this study was the multidisciplinary participation among workgroup members and across survey respondents.

This survey is the first to clearly identify agreement on cSLE research priorities among experts in the field. These results should be used as a roadmap to guide funding opportunities and decisions among lupus stakeholders in both public and private sectors. In the past decade, the overall funding landscape for lupus has been on a decline when comparing fiscal years starting from 2009 to 2019 [24], particularly through the National Institutes of Health (NIH)—the largest public funder of lupus research in the world. In 2017, only 3 out of 248 lupus projects receiving NIH funding included a pediatric focus, and one of those studies was not specific to lupus. This represented only 1.6% of the total government funding towards lupus. Prior fiscal years show similar scarcity in cSLE funded research, and from 2014 to 2016 less than 1% of funds allocated to lupus research were for cSLE-related projects [24].

Given the overall decline in public funding and shockingly low amount of funds specific to cSLE, pediatric rheumatologists and others involved in cSLE care and research are likely more dependent on private funding to conduct ground-breaking research. This research prioritization effort is crucial in showing the drastic and urgent need for additional government funding and re-examining current fund allocations. The research community must also have a coordinated approach to build on promising or new cSLE research based on the top priorities outlined. Respondents clearly identified a need for a unified, multidisciplinary approach to research, which is essential for advancing advocacy and research to support the field caring for and treating children and adolescents with lupus. Future directions include further characterizing top research priorities through a more in-depth survey of respondents who agreed to participate in future follow up work and focus groups of clinicians, investigators, families and patients to understand the alignment of cSLE research goals across groups. Future work may also inform the development of specific requests for applications needed to advance novel research in childhood lupus.

## Conclusions

This multidisciplinary survey effort represents a first research prioritization excerciseeffort among rheumatologists, dermatologists and nephrologists aimed at identifying key research opportunities and improving outcomes for cSLE. The robust participation of members of CARRA, MWPMC and PeDRA allowed for an excellent representation of the priorities of key stakeholders in the care of patients with cSLE. Nephritis and neuropsychiatric disease emerged as key domains with recommended focus on determining best treatments, biomarkers/pathophysiology, drug discovery/novel treatments, refining provider reported clinical outcomes and understanding long term outcomes. Respondents strongly supported the need for multidisciplinary collaboration in cSLE research. Result of this survey will provide a roadmap for future funding and advocacy opportunities for research in cSLE.

## Abbreviations

CARRA: Childhood Arthritis and Rheumatology Research Alliance
cSLE: Childhood onset systemic lupus erythematosus
LN: Lupus nephritis
MWPNC: Midwestern Pediatric Nephrology Consortium
PeDRA: Pediatric Dermatology Research Association
SLE: Systemic lupus erythematosus
